# Comparison of Calculation Methods of Elastic Bonding: Limits of the Gamma Method Using an Example of a Wood–Concrete Composite Floor with Single Loads

**DOI:** 10.3390/ma14237211

**Published:** 2021-11-26

**Authors:** Christian Huber, Karl Deix

**Affiliations:** 1 Camillo Sitte Bautechnikum, HTL Wien 3, A-1030 Vienna, Austria; c.huber@bautechnikum.at; 2 Institute of Material Technology, Building Physics and Building Ecology, University of Technology Vienna, A-1040 Vienna, Austria

**Keywords:** timber–concrete composite floors, static, discrete method

## Abstract

Various methods are available for the calculation of timber–concrete composite floors. The gamma method, which is important in construction practice, as well as the differential equation method, are based on the simplified assumption of a continuous bond between wood and concrete. This makes it possible to analytically calculate the internally statically indeterminate partial section sizes and deformation sizes, analogous to the force size method. In this paper, two typical load situations of concentrated loads (central and off-centre) were analytically and numerically evaluated and compared using the above-mentioned methods (gamma and differential equation), with a discrete method for the case of a timber beam reinforced with a concrete slab using screws as fasteners. The calculation results show significant deviations, which speak for the application of discrete methods in certain load situations and thus limit the usability of the gamma method under certain conditions. For the problem of deflection determination, which is not dealt with in the literature for the discrete method, a numerical method is described in the present work, which was first developed and presented by the first author.

## 1. Introduction

Wood is becoming increasingly important in multi-story residential and office construction as a natural and sustainable building material. In addition to the ecological advantages, the favourable indoor climate and natural appearance of wood surfaces on walls and ceilings are also in the foreground. Here, the wood–concrete composite construction method, which makes optimum use of the mechanical properties of both building materials, has proved to be particularly forward-looking. A large part of the applications of this method is carried out as reinforcement systems for existing wooden ceilings, especially in connection with the overall building stiffness under earthquake loads. For sustainability reasons, wood–concrete composite floors are also increasingly being used in new buildings, especially with the use of CLT (cross-laminated timber) panels.

The lower wooden element, in the form of beams or slabs, mainly carries the bending tensile forces, while the upper continuous concrete slab mainly carries the bending compressive forces. The necessary shear connection is made with various elements, such as bolts, screws, shear collars (cleats), etc. These form a compliant, or elastic, bond between the two elements, which leads to a load-bearing behaviour that lies between no- (loose) and rigid-bond behaviour.

The following is a brief summary of the development of wood–concrete composite floors based on the literature. The first wood–concrete composite floors were developed about 100 years ago, as indicated by Holschemacher et al. in [1], Yeoh et al. in [2] and Grosse et al. in [3]. Design formulas were developed or adopted from Kolbitsch et al. [4] and modified from the “doweled beam theory”. A comparison of the calculation methods was made by Grosse et al. in [5]. The long-term behaviour was modified by Kuhlmann et al. [6] using the gamma method. It was also treated by Grosse et al. [7], Avak et al. [8] and Gerold et al. [9]. Schmidt et al. compared the gamma method with the finite element method in [10] and gave design proposals with graded fastener spacing in [11]. In [12], Rautenstrauch et al. showed a practical design using the framework model. The behaviour of CLT–concrete composite floors with the extended gamma method and the finite element method was investigated by Forsberg et al. in [13], based on the work of Wallner et al. [14].

Essential for load-bearing behaviour are the connecting elements between the wood and concrete. In [15], fully threaded screws were investigated by Heller, and in [16], dowel bars were discussed by Schröter et al. Bonded connections were treated by Schäfers et al. in [17]. Numerical modelling was performed for cleats by Grosse et al. [18], and appropriate models and failure criteria were applied by Schönborn et al., who gave design rules for shear collars in [19]. In one of the newest papers [20], Woschitz et al. described bending tests with CLT and prefabricated concrete plates and compared the calculation methods. In [21], the state-of-the-art of timber–concrete composite structures from cost (European Cooperation in Science and Technology) is described. This documentation forms the basis for the new Eurocode.

For the simplified calculation of wood–concrete composite floors, the strictly spatial system is reduced to an elastically coupled flexural beam system, for which several calculation methods are now available. The differences between the methods result, on the one hand, from the different methods used to generate the static model, and on the other hand, from system-specific conditions (in particular the types of action) that lead to different governing equations.

In this paper, the gamma method according to Möhler [22] and Heimeshoff [23], which is established in practice and relatively easy to calculate, and which is prescribed by Eurocode 5 [24], is compared to and evaluated with the more stringent differential equation method according to Natter and Hoeft [25], as well as the discrete method according to Stüssi [26,27] and Huber [28], on the basis of a representative design situation for selected load cases. This is especially relevant, since the gamma method is known to provide exact, or satisfactorily accurate, results only for symmetrical sinusoidal load situations with an approximately parabolic moment curve.

In reality, when composite screws are used, there is rather a punctual-shear coupling between concrete and wood. The discrete method takes into account the beam sections resulting from the splitting during modelling. It determines the sectional force quantities on a numerical basis, following the finite element method, which is very close to reality.

With a relatively dense distribution of the fasteners, all three of the above methods provide satisfactorily accurate results under uniform load on the single-span beam. However, in the case of single loads on the system or eccentric load location, significant deviations from the valid gamma method can occur. In the case of point load application, which is frequently encountered in construction practice, such as for columns and walls, no evident data are available regarding the accuracy of the gamma method.

## 2. Static System

Since the corresponding values diverge with increasing deviations between the real system and the calculation model, a relatively high single-force application with densely distributed screw spacing was taken as a basis for the calculations, and the uniform load occurring due to self-weight effects was not taken into account.

**Figure 1:**
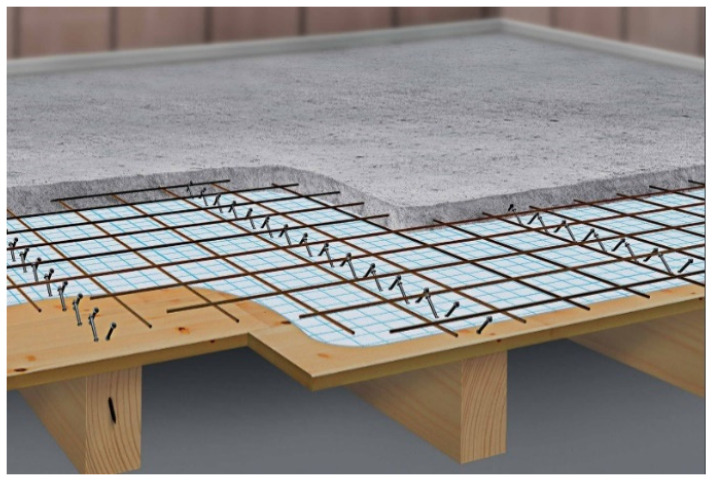
Wood–concrete composite floors—scheme.

**Figure 2:**
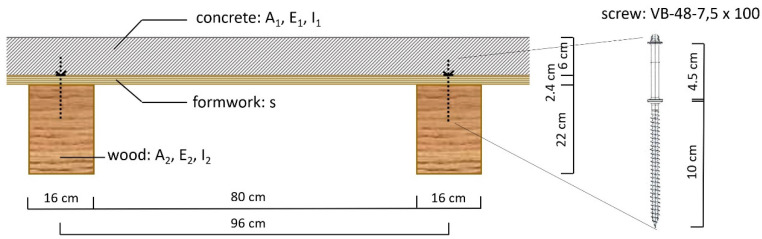
Cross-section dimensions.

**Figure 3:**
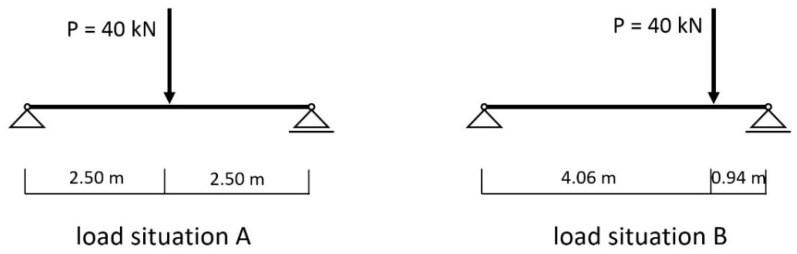
Load situations.

**Figure 4:**
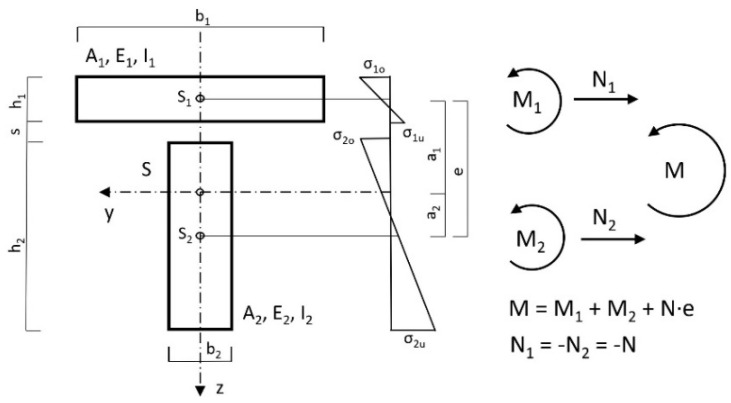
Idealised cross-section with static parameters.

A typical case (Figure 1), in practice, was calculated with the reinforcement of wooden beams by means of a concrete slab. Figure 2, Figure 3 and Figure 4 show the cross-section dimensions, the load situation and the characteristic values; Table 1 contains the characteristic values.

For the “SFS composite screw crossed” fastener, the following displacement moduli were applied according to approval [29]:

K_ser_ = 166 KN/cm for the calculation of the serviceability limit state.

K_u_ = 2/3 … K_ser_ = 111 KN/cm for the calculation of the ultimate limit state.

The screws, arranged crosswise, were anchored with a length of 100 mm in the wood and 45 mm in the concrete. The bolt heads were located in the concrete in the area of the steel reinforcement. The displacement modulus was a compliance coefficient analogous to the modulus of elasticity, where it corresponded to the proportionality factor in the point and to the linear load-displacement law of the fastener in the contact joint direction. In the differential equation method, the joint stiffness (K) is also important. This was calculated as the quotient of the displacement modulus K (K_ser_ for the deformations and K_u_ for the forces) divided by the fastener spacing e’ (displacement modulus notionally smeared over the bolt spacing). The fastener spacing in the longitudinal direction of the beam was e’ = 11.1 cm, which resulted from 45 sections for the span of 500 cm.

## 3. Theory and Calculation

### 3.1. Calculation of Force Quantities with the Discrete Method

**Figure 5:**
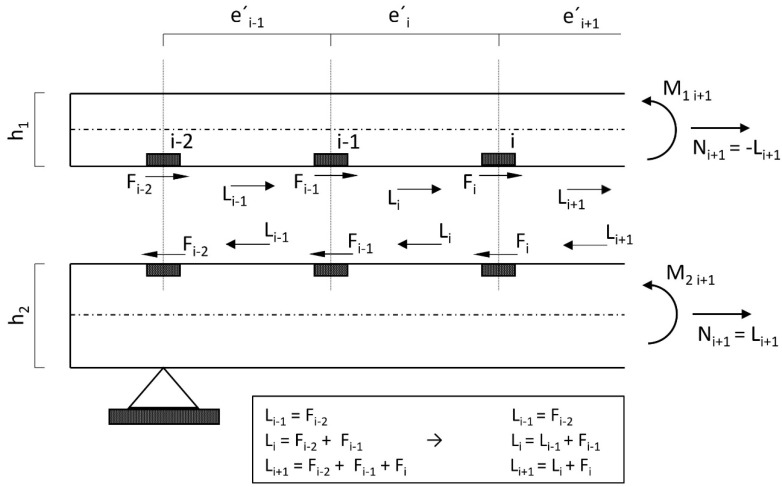
Exposed composite beam with cutting forces.

In the method first presented by Stüssi [26,27], the fasteners were represented on the idealised structural model as point-acting single dowels with linear elastic deformation laws (transverse to the joint). Likewise, linear material laws and the validity of the Bernoulli hypothesis were assigned to the concrete belt bar and the timber beam for the individual cross-sections (but not for the total cross-section). Thus, there was an internal highly statically indeterminate structure, with the excess dowel forces acting horizontally in the connection joint (Figure 5). For further consideration, the system was split up and, according to the superposition principle, superimposed on the composite-free girder—each loaded with the dowel forces (acting in the gravity line of the contact joint) and the external load.

L_i_ corresponds to the dowel-force resultant, calculated field by field from the support. The relationship between the dowel-force resultant and the section-normal force is shown in Figure 4. In the belt, for equilibrium reasons, N_i_ = −L_i_, while in the web N_i_ = L_i_, applies. Using the main bending equation and the material law, length changes in the contact joint due to the affected dowel-force resultants and partial moments (respectively due to the external load and due to the dowel forces) can be calculated.

#### 3.1.1. Partial Moments Due to the External Load

Due to the relatively simple conditions at the unbonded beam, the partial moments due to the external load can be expressed by the total moment M. Following Figure 4, the absence of partial-normal forces means that (N_1_ = N_2_ = 0) is valid for the unbonded beam for this load case:

(1) $$M={\;M}_{1}+{\;M}_{2}$$

Due to the equal deflections of belt and web (effects due to the twisting of the cross-section were not taken into account), whereby for any beam location w_1_ = w_2_, after differentiating twice and neglecting the shear deformation, the geometric compatibility condition follows this equation:

(2) $$\frac{M_{1}}{E_{1}\;\cdot {\;I}_{1}}=\frac{M_{2}}{E_{2}\;\cdot {\;I}_{2}}\;$$

Thus, two equations are available for the two unknowns (M_1_ and M_2_), and the task can be solved mathematically and unambiguously if the total moment M is known, whereby the partial moments can be expressed by the total moment. The total moment M_0m,_ averaged over the section, was used to determine the elongation changes.

#### 3.1.2. Partial Moments According to Dowel Forces

The partial moment curve due to the dowel forces is constant in sections.

#### 3.1.3. Compatibility Condition

**Figure 6:**
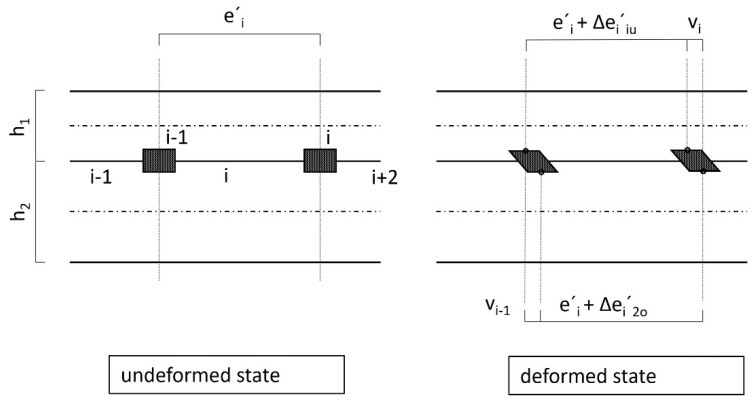
Continuity condition in the contact joint.

The fulfilment of the constraint condition, according to Figure 6 and Equation (3), leads directly to Equation (4), which can be set up for each field.

In this case:

e_i_’ + Δe_i_’_1u_ + v_i_ = e_i_’ + Δe_i_’_2o_ + v_i−1_ ⇒      Δe_i_^’^_1u_ + v_i_ = Δe_i_^’^_2o_ + v_i−1_ (3)

e_i_’: Centre distance of dowels in section i in the undeformed or unloaded state.

Δe_i_’_1u_: Length change of the distance e_i_’ of the dowels at the height of the lower edge fibre of the upper beam 1 due to the forces and moments acting in this section (moments are to be averaged).

v_i_: Deformation of dowel i in the direction of the contact joint due to dowel force F_i._

Δe_i_’_2o_: Change in the length of the distance e_i_’ of the dowels at the level of the upper edge fibre of the lower beam 2 due to forces and moments acting in this section.

v_i−1_: Deformation of the dowel i-1 in the direction of the contact joint due to the dowel force F_i−1_.

(4) $$-L_{i-1}+L_{i}\cdot \left[2+K\cdot e_{i}^{\prime }\cdot \left(\frac{h_{1}^{2}}{4\cdot E_{1}\cdot I_{1}}+\frac{h_{2}^{2}}{4\cdot E_{2}\cdot I_{2}}+\frac{1}{E_{1}\cdot {\;A}_{1}}+\frac{1}{E_{2}\cdot {\;A}_{2}}\right)\right]-L_{i+1}=\frac{M_{0\mathrm{im}}\cdot K\cdot e^{\prime }\cdot \left(h_{1}+h_{2}+2\cdot s\right)}{2\cdot \left(E_{1}\cdot I_{1}+E_{2}\cdot I_{2}\right)}$$

where M_0im_ is the mean total moment in section i. With 45 sections, this results in a system of equations with 45 equations and 45 unknown dowel-force resultants (L_i_) or partial-normal forces (N_i_).

In matrix notation, it follows:

$$\left(\begin{array}{cccccccccc} c & -1 & 0 & 0 & . & . & 0 & 0 & 0 & 0\\ -1 & c & -1 & 0 & . & . & 0 & 0 & 0 & 0\\ 0 & -1 & c & -1 & . & . & 0 & 0 & 0 & 0\\ 0 & 0 & -1 & c & . & . & 0 & 0 & 0 & 0\\ . & . & . & . & . & . & . & . & . & .\\ . & . & . & . & . & . & . & . & . & .\\ 0 & 0 & 0 & 0 & . & . & c & -1 & 0 & 0\\ 0 & 0 & 0 & 0 & . & . & -1 & c & -1 & 0\\ 0 & 0 & 0 & 0 & . & . & 0 & -1 & c & -1\\ 0 & 0 & 0 & 0 & . & . & 0 & 0 & -1 & c \end{array}\right)\left(\begin{array}{c} L_{1}\\ L_{2}\\ L_{3}\\ L_{4}\\ .\\ .\\ L_{42}\\ L_{43}\\ L_{44}\\ L_{45} \end{array}\right)=\;\left(\begin{array}{c} m_{1}\\ m_{2}\\ m_{3}\\ m_{4}\\ .\\ .\\ m_{42}\\ m_{43}\\ m_{44}\\ m_{45} \end{array}\right)$$

In the matrix, the diagonal value c corresponds to the constant bracket expression on the left side in Equation (4). The factor c is independent of the load, and depends on, among other things, the displacement modulus. The right column vector of the equation system (m_1_ to m_45_) also includes the load-case-dependent total moment curve (m_i_) of the bondless basic system. Due to the special problem definition, with two load cases and one displacement modulus each for the load-bearing capacity and the deformations, a total of four equation systems must be solved.

**Figure 7:**
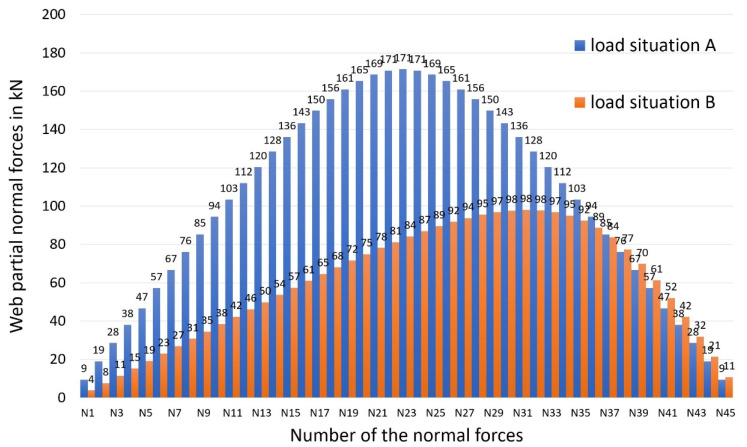
Web partial-normal forces for the load-bearing capacity.

The numerical evaluations of the four possible combination cases lead to partial-normal forces in the joint. Figure 7 shows this for load-bearing capacity (the shape for the calculation of the deformations is similar). These correspond to the dowel-force resultants, accordingly sign-weighted as already described (belt: −, web: +). In the case of load situation A, the numerically calculated dowel-force resultants/partial-normal forces of the individual beam sections were distributed symmetrically over the beam length, whereas in load situation B, they were distributed asymmetrically due to the different partial moment distribution.

Furthermore, the partial moments for the real system were obtained directly from this in accordance with Figure 5, with compound use of the following equations (Superposition principle):

(5) $$M_{1}=\frac{M\cdot E_{1}\cdot I_{1}}{E_{1}\cdot I_{1}+E_{2}\cdot I_{2}}-N_{i}\cdot \left(\frac{h_{1}+s}{2}\right)$$

(6) $$M_{2}=\frac{M\cdot E_{2}\cdot I_{2}}{E_{1}\cdot I_{1}+E_{2}\cdot I_{2}}-N_{i}\cdot \left(\frac{h_{2}+s}{2}\right)$$

where s is the thickness of the formwork according to Figure 2. The results are summarised for the force application point (in the centre of the field for load situation A and 0.94 m distance from the right bearing point for load situation B) in Table 2.

### 3.2. Derivation of Deformations for the Discrete Method

In ref. [28], a proposal for the determination of deflections based on the “principle of virtual forces” was developed for the discrete method. Here again, the “superposition principle” was used as previously described.

According to the working principle, the auxiliary system (one-system) was again the unbonded beam with a load of 1 at the point and in the direction where the deflection was sought.

The respective moment curves in the belt and web were then to be superimposed according to Equation (7), whereby the deflection components due to shear forces were neglected.

(7) $$w=\int^{\;}\frac{M\cdot \;\bar{M}}{E\cdot I}\cdot \mathrm{dx}$$

**Figure 8:**
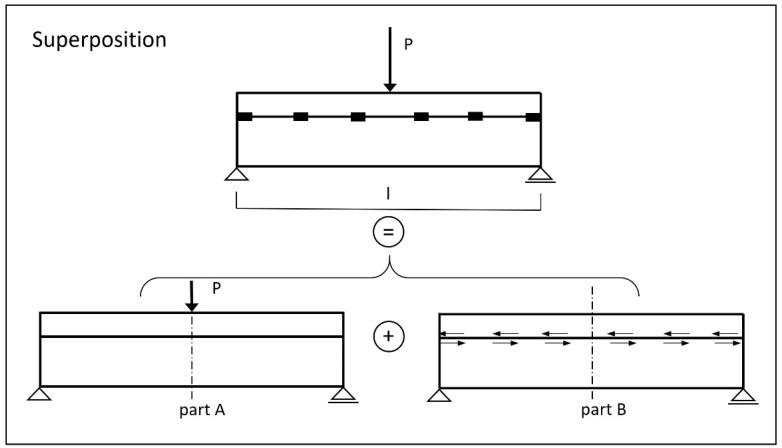

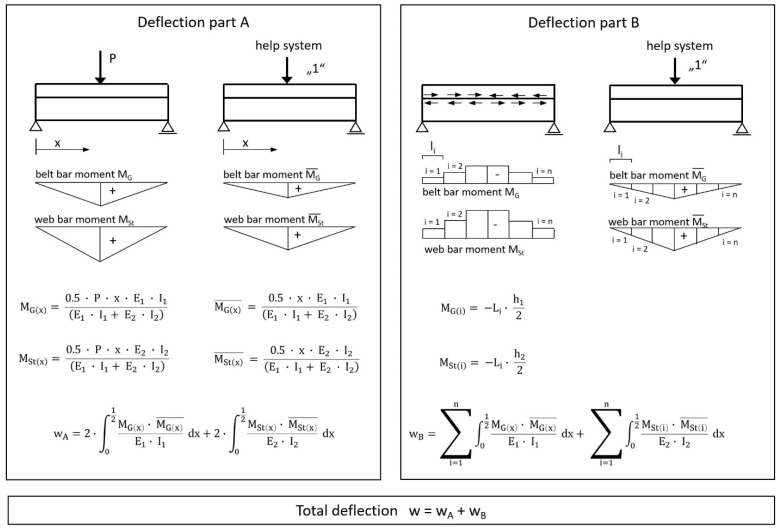
Procedure for determining deflections with principle of virtual forces.

The following Figure 8 illustrates the procedure using the example of a single-span beam with a point load in the centre of the span. For the total deflection, the individual deflection components must be added.

The deflection at the point of force application (centre of the field for load situation A) resulted in 1.66 cm for load situation A and 0.69 cm for eccentric-load situation B, using the method described and including the numerically determined partial-normal force results according to Figure 8.

### 3.3. Calculation of Force Quantities and Deformations with the Differential Equation Method

The derivation and solution of the differential equations were carried out by Natterer and Hoeft [25]. The basis for the derivation of the required additional governing equations for the internally statically indeterminate problem (again assuming linear material laws for the composite partners, including the connecting means and the validity of the flatness of the partial cross-sections after bending) was again the fulfilment of the constraint condition in the contact joint of an infinitesimal beam element (dx) with a continuous connection between concrete and wood. This leads to a differential equation system for the contact joint displacement (u) and the beam deflection (w). In a publication [25] of March 1987, the differential equation system was solved for the most common loading situations, leading to continuous governing equations for the mechanical parameters.

**Figure 9:**
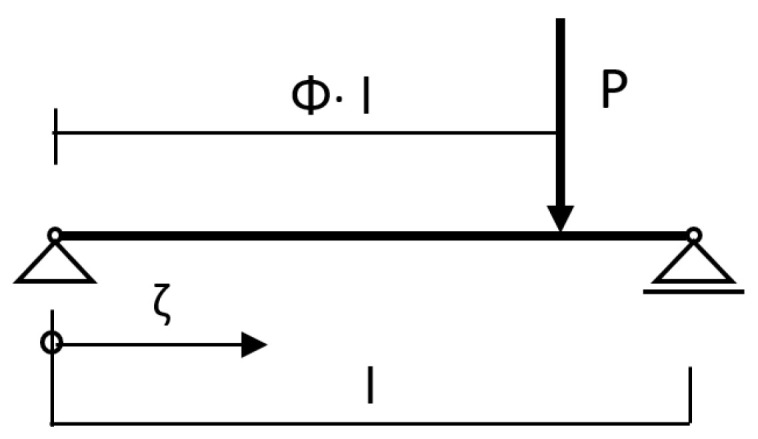
Load situation.

In the following, the main equations and preliminary values for the calculation of a single-span beam with concentrated load are presented, where Φ indicates the location of the concentrated load with respect to the span (Figure 9).

For the load position (single load at the centre of the field), Φ = 0.5, and the location of the sought mechanical quantities can be expressed by the related bar length variable ζ = 0.5.

In order to simplify the application of the equations of determination, the following preliminary values must be determined (Figure 4 and Table 1 are valid) according to [25]:

(8) $$\lambda^{2}=\left(k\cdot \frac{\left(E_{1}\cdot A_{1}+E_{2}\cdot A_{2}\right)}{E_{1}\cdot A_{1}\cdot E_{2}\cdot A_{2}}+\frac{k\cdot e^{2}}{E_{1}\cdot I_{1}+E_{2}\cdot I_{2}}\right)\cdot l^{2}$$

(9) $$\frac{a^{2}}{1-a^{2}}=\frac{E_{1}\cdot A_{1}\cdot E_{2}\cdot A_{2}\cdot e^{2}}{\left(E_{1}\cdot A_{1}+E_{2}\cdot A_{2}\right)\cdot \left(E_{1}\cdot I_{1}+E_{2}\cdot I_{2}\right)}$$

(10) $$a^{2}=\frac{1}{\frac{\left(E_{1}\cdot A_{1}+E_{2}\cdot A_{2}\right)\cdot \left(E_{1}\cdot I_{1}+E_{2}\cdot I_{2}\right)}{E_{1}\cdot A_{1}\cdot E_{2}\cdot A_{2}\cdot e^{2}}+1}$$

(11) $$B=E_{1}\cdot I_{1}+E_{2}\cdot I_{2}+\frac{E_{1}\cdot A_{1}\cdot E_{2}\cdot A_{2}\cdot e^{2}}{\left(E_{1}\cdot A_{1}+E_{2}\cdot A_{2}\right)}$$

(12) $$c=\frac{E_{1}\;\cdot I_{1}}{E_{1}\cdot {\;I}_{1}+{\;E}_{2\;}\cdot {\;I}_{2}}$$

(13) $$d=\frac{E_{2}\;\cdot I_{2}}{E_{1}\cdot {\;I}_{1}+{\;E}_{2\;}\cdot {\;I}_{2}}$$

The normal force and the bending moments result in:

(14) $$N=P\cdot l\cdot \frac{a^{2}}{e}\cdot \left[\left(1-\Phi \right)\cdot \zeta -\frac{1}{\lambda }\cdot \frac{\sin h\left[\lambda \cdot \left(1-\Phi \right)\right]}{\sin h\left(\lambda \right)}\cdot \sin h\left(\lambda \cdot \zeta \right)\right]$$

(15) $$M_{1}=P\cdot l\cdot d\cdot \left[\left(1-a^{2}\right)\cdot \left(1-\Phi \right)\cdot \zeta +a^{2}\cdot \frac{1}{\lambda }\cdot \frac{\sin h\left[\lambda \cdot \left(1-\Phi \right)\right]}{\sin h\left(\lambda \right)}\cdot \sin h\left(\lambda \cdot \zeta \right)\right]$$

(16) $$M_{2}=P\cdot l\cdot c\cdot \left[\left(1-a^{2}\right)\cdot \left(1-\Phi \right)\cdot \zeta +a^{2}\cdot \frac{1}{\lambda }\cdot \frac{\sin h\left[\lambda \cdot \left(1-\Phi \right)\right]}{\sin h\left(\lambda \right)}\cdot \sin h\left(\lambda \cdot \zeta \right)\right]$$

Table 3 summarises the results of the calculations.

To illustrate and better classify the results, the fictitious case of a rigid bond between wood and concrete was also calculated using this method, and the results are presented in Table 4.

According to [25], the deflections were given by Equation (17) for load situation A as 1.68 cm and for load situation B as 0.71 cm. In comparison, the deflections under the assumption of a rigid composite would be calculated as 1.07 cm for load situation A and 0.40 cm for load situation B.

(17) $$w=\frac{P\cdot l^{3}}{B}\cdot \left\{\frac{a^{2}}{1-a^{2}}\left[\frac{1}{\lambda^{2}}\cdot \left(1-\Phi \right)\cdot \zeta -\frac{1}{\lambda^{3}}\cdot \frac{\sin h\left[\lambda \cdot \left(1-\Phi \right)\right]}{\sin h\left(\lambda \right)}\cdot \sin h\left(\lambda \cdot \zeta \right)\right]+\frac{1}{6}\cdot \left(1-\Phi \right)\cdot \left(2\cdot \Phi -\Phi^{2}-\zeta^{2}\right)\cdot \zeta \right\}$$

### 3.4. Gamma Method

Usually, the gamma method is used for composite structures. This is a simple calculation method, which is also the standard method in Eurocode 5 [24]. The advantage lies in compact and easy-to-use formulas, whereby the effective bending stiffness of the entire beam can be used for deformation calculations. With the introduction of an effective moment of inertia, the requirement that the curvature of the individual parts must correspond to the curvature of the entire beam is again met for the composite-free case in an extended sense.

(18) $$\frac{M_{1}}{E_{1}\;\cdot {\;I}_{1}}=\frac{M_{2}}{E_{2}\;\cdot {\;I}_{2}}=\frac{M}{E_{v}\;\cdot {\;I}_{\mathrm{eff}}}\;$$

The comparative elastic modulus E_v_ can be free selected; mostly, E_v_ = E_2_ is selected.

(19) $$M={\;M}_{1}+{\;M}_{2}$$

Thus, for the effective moment of inertia after some transformation

(20) $$I_{\mathrm{eff}}=\frac{E_{1}}{E_{V}}\cdot I_{1}+\frac{E_{2}}{E_{V}}\cdot I_{2}$$

In the case of a rigid compound, the Steiner component also appears. In the compound-less case (no Steiner part is effective), this corresponds to a weight of the Steiner part of 0. All cases of the real compound can therefore be classified between these two cases (with gamma as a weighting factor = 0 to 1).

The γ-value was obtained by comparing the terms of the gamma method with those of the differential equation method. Compact terms were obtained only for sinusoidal loads, but large and complicated terms were obtained for uniform loads and symmetrically concentrated loads. According to [3], the following formulas result for the sinusoidal load:

(21) $$f=\frac{\pi^{2}\cdot E_{1}\cdot A_{1}\cdot \;e\;\prime }{l^{2}\cdot K}$$

(22) $$\gamma =\frac{1}{1+f}$$

(23) $$a_{2}=\frac{1}{2}\cdot \frac{\gamma \cdot E_{1}\cdot A_{1}\cdot \left(h_{1}+h_{2}+2\cdot s\right)}{\left(\gamma \cdot E_{1}\cdot A_{1}+E_{2}\cdot A_{2}\right)}$$

(24) $$a_{1}=\frac{1}{2}\cdot \left(h_{1}+h_{2}+2\cdot s\right)-a_{2}$$

where f is an auxiliary value. With the arbitrary comparative elasticity modulus E_v_, the effective moment of inertia can be calculated:

(25) $$I_{\mathrm{eff}}=\frac{E_{1}}{E_{V}}\cdot I_{1}+\frac{E_{2}}{E_{V}}\cdot I_{2}+\gamma \cdot \frac{E_{1}}{E_{V}}\cdot A_{1}\cdot a_{1}{}^{2}+\frac{E_{2}}{E_{V}}\cdot A_{2}\cdot a_{2}{}^{2}$$

For the total moment for load situation A:

$$M=\frac{40\;\cdot \;5}{4}=50.0\;\mathrm{kNm}$$

and for load situation B:

$$M=\frac{40\cdot 4.056\cdot 0.944}{5}=30.631\;\mathrm{kNm}$$

The partial moments M_1_ and M_2_, as well as the normal force, result in

(26) $$M_{1}=\frac{M}{E_{V}\cdot I_{\mathrm{eff}}}\cdot E_{1}\cdot I_{1}$$

(27) $$M_{2}=\frac{M}{E_{V}\cdot I_{\mathrm{eff}}}\cdot E_{2}\cdot I_{2}$$

(28) $$N=\frac{M}{E_{V}\cdot I_{\mathrm{eff}}}\cdot E_{2}\cdot A_{2}\cdot a_{2}$$

This results in the values compiled in Table 5:

The deflections can be determined with the conventional formulas of structural analysis, namely with the effective bending stiffness according to the gamma method in the denominator. Load situation A for x = l/2 follows:

(29) $$w=\frac{F\cdot {\;l}^{3}}{48\cdot E_{v}\cdot I_{\mathrm{eff}}}$$

and for the load situation B for x = 0.94

(30) $$w=\frac{F\cdot {\;a}^{2}\cdot b^{2}}{3\cdot E_{v}\cdot I_{\mathrm{eff}}\cdot l}$$

This results in 1.65 cm for load situation A and 0.62 cm for load situation B.

## 4. Comparison of the Results

**Figure 10:**
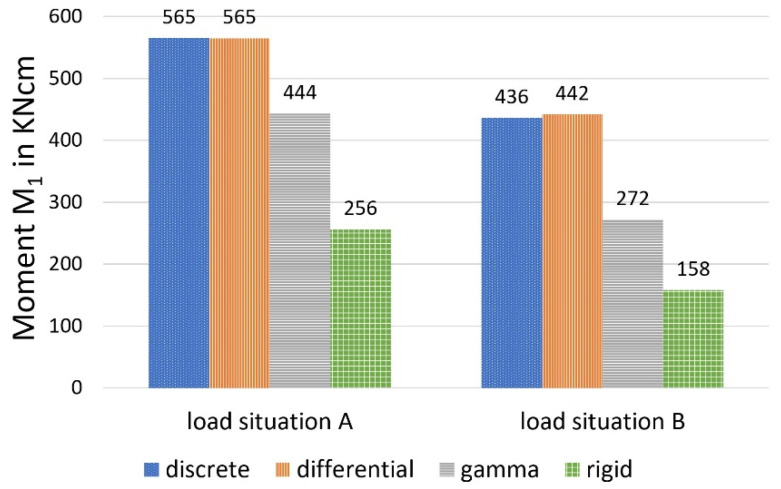
Moment M_1_, calculated with different methods.

**Figure 11:**
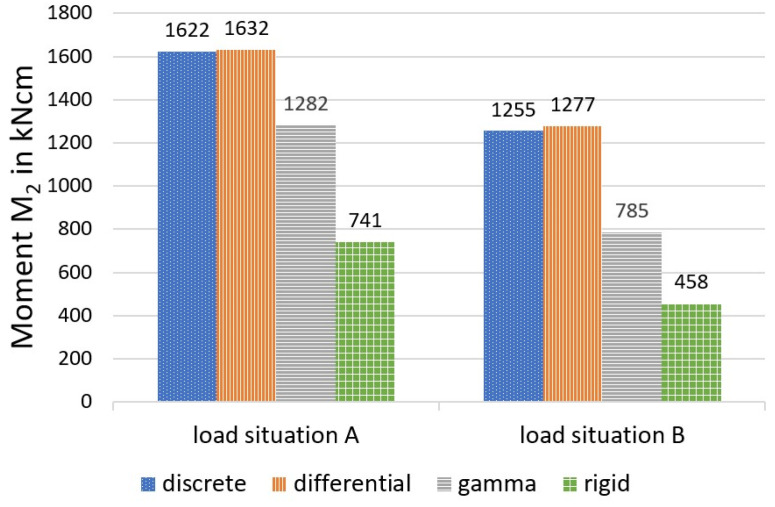
Moment M_2_, calculated with different methods.

**Figure 12:**
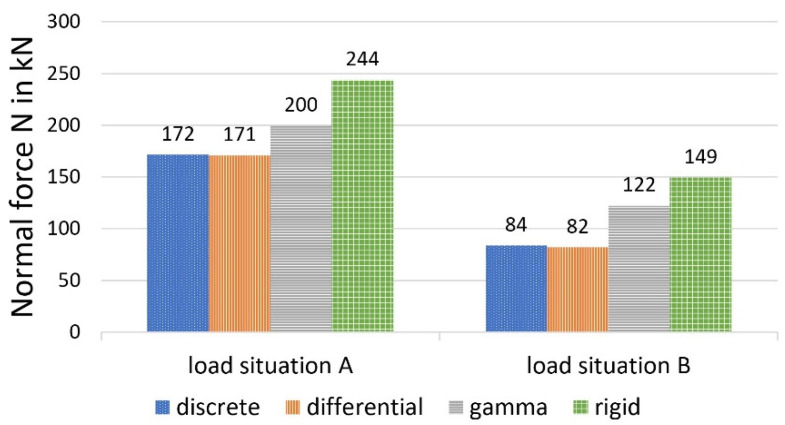
Normal force N, calculated with different methods.

**Figure 13:**
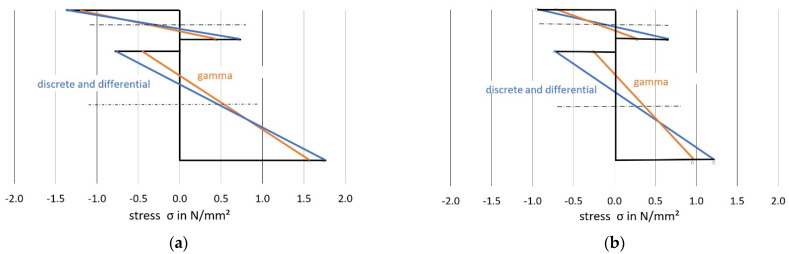
Longitudinal stresses for load situation A (**a**) and load situation B (**b**).

Figure 10, Figure 11 and Figure 12 show a comparison of the bending moments M_1_ and M_2_ and the normal forces N, and Figure 13 shows the stresses.

It should first be noted that, under the given conditions, the differential equation method and the discrete method provide almost identical values for the selected load-bearing behaviour variables. This circumstance can be explained by the relatively dense arrangement of the bolt pairs, which creates an almost continuous bond between the wood and the concrete. The sectionally constant moments and normal forces of the discrete method deviate only slightly from the continuous lines of the differential equation method at small distances (here, 45 fields). In contrast to screws, which are always arranged relatively closely, larger distances are present in the case of cleats. In [20], comparative calculations with cleats, which formed only 10 sections (and single loads), were carried out, and also showed deviations between the methods.

As shown in the comparative calculation, the gamma method underestimates more accurate methods with respect to stress determination for both load situations. For load situation A, the gamma method calculates a difference of about minus 13 per cent for σ_1o_ (based on the values calculated with the more stringent method) and minus 11 per cent for σ_2u_. For load situation B, the differences were even greater (minus 25 per cent for the upper stress versus minus 21 per cent for the lower stress). 

This can be explained primarily by a redistribution of the internal forces in the direction of the rigid composite, which was more pronounced in load situation B, according to the data available here. Use of the gamma method leads to a reduction of around 22 per cent for load situation A (minus 39 per cent in load situation B) in the case of the two partial moments, compared with the more stringent methods, with a simultaneous increase in the partial-normal-force stress of 18 per cent (plus 49 per cent in load situation B). This allows the conclusion that, in the case of the central concentrated load and with increasing deviation from this, the gamma method underestimates the bending stress and overestimates the partial-normal-force stress. The values, therefore, erroneously approach the exact results assuming a rigid composite. This was also ultimately reflected in the deflections (in particular load situation B, with minus 11 per cent). 

**Figure 14:**
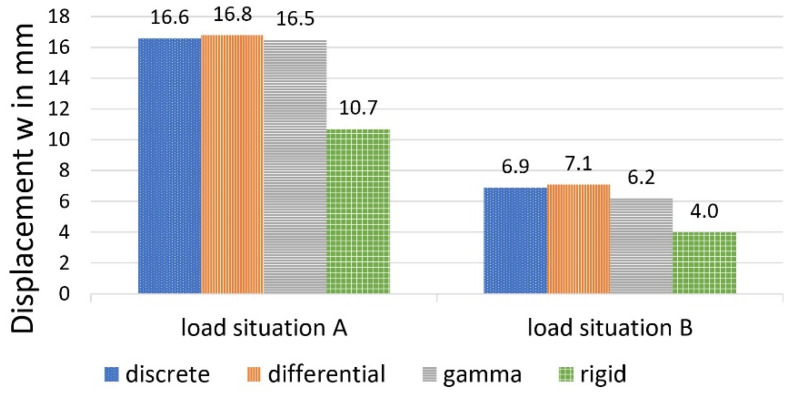
Deflection w, calculated with different methods.

The deflections, shown in Figure 14, are approximately the same for all 3 calculation models and larger than with the rigid system.

## 5. Conclusions

In summary, the gamma method is to be considered as a model-rate inaccurate method for the computational determination of two-part wood–concrete hybrid beams with common design features, especially for predominantly eccentrically located load situations compared to the more stringent method.

The gamma method underestimates the stress and deformation quantities to be determined for both the ultimate limit state design and the serviceability check, and is therefore on the unsafe side.

From a design point of view, the differences can, in any case, become decisive with regard to an exact verification, and thus represent a design-relevant criterion under certain conditions. Deviations can also occur with a larger spacing of the cleats—even with uniform loads—as described in [20]. In view of these results, it is therefore recommended to use the more stringent differential equation method for mathematical prediction in such exceptional situations.

In summary, it is to be stated that the discrete method, which involves a high computational effort, is a valuable methodology for the accurate simulation of conventional wood–concrete composite beams, especially in the case of single loads.

## Figures and Tables

**Table 1:** Characteristic values.

Plate		Beam	
Concrete	C20/25	Wood	C22
Elastic modulus E_1_	3000 kN/cm^2^	Elastic modulus E_2_	1000 kN/cm^2^
Thickness h_1_	6 cm	Beam high h_2_	22 cm
Contributing plate width b_1_	91 cm	Beam width b_2_	16 cm
Area A_1_	546 cm^2^	Area A_2_	352 cm^2^
Moment of Inertia I_1_	1638 cm^4^	Moment of Inertia I_2_	14,197 cm^4^
Centre of mass distance e	16.4 cm	Thickness of the formwork s	2.4 cm

**Table 2:** Results according to the discrete method.

Load Situation	M_1_	M_2_	N	σ_1o_	σ_2u_
A	565 kNcm	1622 kNcm	171.5 kN	−1.35 kN/cm^2^	1.74 kN/cm^2^
B	436 kNcm	1255 kNcm	83.7 kN	−0.95 kN/cm^2^	1.21 kN/cm^2^

**Table 3:** Results according to the differential equation method.

Load Situation	M_1_	M_2_	N	σ_1o_	σ_2u_
A	565 kNcm	1632 kNcm	171.0 kN	−1.35 kN/cm^2^	1.75 kN/cm^2^
B	442 kNcm	1277 kNcm	82.0 kN	−0.96 kN/cm^2^	1.22 kN/cm^2^

**Table 4:** Results for the rigid bond.

Load Situation	M_1_	M_2_	N	σ_1o_	σ_2u_
A	256 kNcm	741 kNcm	244.1 kN	−0.92 kN/cm^2^	1.27 kN/cm^2^
B	158 kNcm	458 kNcm	149.2 kN	−0.56 kN/cm^2^	0.78 kN/cm^2^

**Table 5:** Results according to the gamma method.

Load Situation	M_1_	M_2_	N	σ_1o_	σ_2u_
A	444 kNcm	1282 kNcm	199.7 kN	−1.18 kN/cm^2^	1.56 kN/cm^2^
B	272 kNcm	785 kNcm	122.3 kN	−0.72 kN/cm^2^	0.96 kN/cm^2^

## Data Availability

Not applicable.
